# Supplementation with Zn-loaded montmorillonite enhanced Zn ion transport, trace element deposition, antioxidant capacity, and intestinal function in broilers

**DOI:** 10.3389/fvets.2025.1609339

**Published:** 2025-06-13

**Authors:** Shizhen Qin, Haibo Wang, Shijiao Qin, Jinlv Li, Defu Tang, Zhaoguo Shi

**Affiliations:** ^1^College of Animal Science and Technology, Gansu Agricultural University, Lanzhou, China; ^2^Lanzhou Qilihe District Agricultural Technology Extension Station, Lanzhou, China

**Keywords:** broilers, Zn-loaded montmorillonite, antioxidant capacity, zinc transporter, intestinal function

## Abstract

This study aimed to investigate the effects of dietary zinc-loaded montmorillonite (Zn-MMT) on performance, Zn transporter expression, metal deposition, antioxidant capacity, and intestinal function in broilers. A total of 144 one-day-old male Cobb broilers were randomly divided into three treatment groups. The broilers in the control group (CK) were fed a corn–soybean meal basal diet, while the experimental groups were fed a basal diet supplemented with 40 mg/kg Zn-MMT and ZnSO_4_ (in terms of Zn content). The results showed that Zn-MMT had no significant (*p* > 0.05) effect on average daily gain (ADG), average daily feed intake (ADFI), or carcass parameters, but it significantly (*p* < 0.05) reduced the feed-to-gain (F: G) ratio. Dietary Zn supplementation increased (*p* < 0.05) the expression of Zn transporter 1 (*ZnT-1*), Zn transporter 5 (*ZnT-5*), metallothionein (*MT*), and *MTF-1* mRNA in the jejunum and the Zn content in the tibia and whole blood. In addition, it increased (*p* < 0.05) total antioxidant capacity (T-AOC) and Cu/Zn-SOD while reducing (*p* < 0.05) malondialdehyde (MDA) levels in the liver and jejunum. However, no significant effect (*p* > 0.05) was observed on the microbial population in the cecum. Furthermore, compared to the CK and ZnSO_4_ groups, Zn-MMT significantly (*p* < 0.05) increased the mRNA expression of *MT-3* and divalent metal transporter 1 (*DMT-1*) in the jejunum and promoted the storage of Zn in the liver and pancreas. It also significantly (*p* < 0.05) increased villus height (VH) and the villus heightto-crypt depth (VH/CD) ratio in the duodenum and jejunum, increased the VH/CD ratio in the ileum, and reduced CD in the duodenum. In conclusion, supplementation with Zn-MMT in a corn–soybean meal basal diet can increase the expression of metal transporters, promote Zn deposition, enhance antioxidant capacity, improve intestinal tissue parameters, and increase Zn utilization.

## Introduction

Zinc (Zn), an essential trace mineral widely utilized in poultry nutrition, plays a critical role in avian physiological processes and survival (1). Functioning as a structural component or enzymatic cofactor, Zn actively participates in over 300 metalloenzymes and functional proteins that regulate fundamental biological processes in broilers (2). From a nutritional perspective, Zn demonstrates multifaceted functionality in poultry systems, including enhanced nutrient assimilation, antioxidant defense mechanisms, and the maintenance of homeostasis (3, 4). As the second most abundant trace mineral in avian organisms, Zn exerts regulatory control over physiological pathways governing growth performance, immune competence, and reproductive efficiency in broilers (5, 6). The significance of this mineral extends to its pivotal role in antioxidant systems and immunomodulatory responses, as evidenced by recent studies on broilers (7–9). Notably, Zn interacts competitively with other trace elements, including copper (Cu), iron (Fe), and manganese (Mn), during intestinal absorption (10). Experimental evidence indicates that Zn serves as the first limiting element in these interactions, with its deficiency significantly impairing the absorption and utilization of Fe, Mn, and Cu (11). Clinical manifestations of Zn deficiency in poultry encompass growth retardation, immunosuppression, and increased susceptibility to disease. However, broilers lack the ability for endogenous Zn synthesis, making them entirely dependent on dietary sources to meet their physiological requirements. Consequently, Zn supplementation in poultry diets is essential to meet nutritional requirements and support optimal growth. Among inorganic Zn sources, zinc sulfate (ZnSO₄) remains the predominant additive in commercial formulations. Nevertheless, studies have demonstrated that inorganic Zn compounds exhibit low bioavailability in broilers (12), potentially limiting their metabolic utilization. Recent studies have reported that organic manganese compounds, such as manganese amino acid chelates, exhibit enhanced manganese utilization efficiency in animal nutrition. However, their industrial application in feed additives remains limited due to stringent production processes and elevated manufacturing costs, which impose significant barriers to widespread adoption. Therefore, structural modification of ZnSO₄ (e.g., through chelation or encapsulation) and the implementation of sustained-release delivery systems targeting distal intestinal segments may offer viable strategies to enhance Zn absorption efficiency.

Montmorillonite (MMT), a naturally abundant silicate clay mineral, is characterized by a 2:1 layered structure, with silica tetrahedral sheets sandwiched between alumina octahedral sheets. This unique configuration endows MMT with exceptional physicochemical properties, including a high specific surface area, strong ion exchange capacity, superior adsorption capability, and excellent colloidal stability. Notably, these attributes, particularly its expansive surface area, remarkable adsorption capacity, and chemical inertness, have established MMT as an effective carrier for the controlled delivery of mineral elements. Compared to traditional metallic supplements, metal-loaded montmorillonite demonstrates superior characteristics in terms of enhanced safety profile, sustained efficacy, and improved biological activity. Substantial evidence suggests that the bioavailability of trace mineral elements in metal-intercalated montmorillonite formulations significantly exceeds that of conventional inorganic mineral sources (13, 14). Controlled studies have shown that dietary supplementation with Zn oxide-modified clay in weaned piglets (15) and broiler chickens (16) enhances growth performance and promotes intestinal functional development. Particularly, Wang et al. (17) demonstrated that Zn-bearing palygorskite and Zn-bearing clinoptilolite effectively inhibit *Escherichia coli* proliferation while reducing its intestinal colonization density in broilers. Our preliminary investigations have revealed that Zn-loaded montmorillonite (Zn-MMT) exerts more pronounced positive effects on broiler growth parameters compared to inorganic Zn supplements. This experimental observation corroborates previous studies, demonstrating the superior bioavailability of Zn-MMT compared to ZnSO₄ in avian nutritional applications. Therefore, we hypothesized that Zn-MMT could serve as a potential alternative to inorganic Zn in broiler nutrition through multiple mechanisms: (1) enhancing Zn ion transport efficiency; (2) optimizing trace element deposition patterns; (3) improving systemic antioxidant capacity; and (4) reinforcing intestinal barrier function. To validate this hypothesis, the current study was designed to systematically evaluate the impacts of Zn-MMT supplementation on broiler growth performance, zinc transport dynamics, and trace element deposition.

## Materials and methods

### Ethics statement

The animal experiments were conducted in accordance with the ARRIVE guidelines and were approved by the Institutional Animal Care and Use Committee (IACUC) of Gansu Agricultural University (Protocol No. GAU-Eth-AST-2024-026). All procedures involving animals adhered to the National Research Council’s Guide for the Care and Use of Laboratory Animals, with special confirmation that no specimens from threatened or protected species were employed throughout the study.

### Zn-loaded montmorillonite

Zn-MMT was provided by the company, Chifeng and Mingsheng Chemical Co., LTD., and was determined by the testing company, Lanzhou Zhongjike Test Technology Co., LTD. The Zn content was 10,408.80 mg/kg.

### Experimental design, animals, and diet groups

A total of 144 one-day-old male Cobb broilers with uniform initial body weight (36.5 ± 0.3 g) were obtained from Huaqin Agriculture and Animal Husbandry Technology Co., Ltd. (Shaanxi, China). The birds were randomly allocated to three dietary treatment groups using a completely randomized design, with six replicates per group and eight birds per replicate. The experiment was conducted over a 42-day period under controlled environmental conditions. All birds were housed in wire-floored cages with continuous access to mash feed and water.

The temperature was maintained at 34–35°C during the first 3 days post-hatching, followed by a gradual weekly reduction of 2–3°C until reaching 22°C by the end of the trial.

The treatments were as follows: (1) the control, fed a corn–soybean meal basal diet (CK); (2) the basal diet supplemented with 40 mg/kg Zn as Zn-MMT; and (3) the basal diet supplemented with 40 mg/kg Zn as ZnSO_4_. In both cases, Zn-MMT and ZnSO_4_ were adjusted to provide the same zinc content by replacing the carrier. The formulation and calculated nutrient levels of the basal diet are shown in Table 1.

**Table 1:** The formulation and calculated nutrient levels of the broiler basal diet (%).

Items	Days1 ~ 21	Days22 ~ 42	Items	Days1 ~ 21	Days22 ~ 42
Ingredients			Nutrient levels^D^		
Corn	52.39	56.20	Metabolic Energy (MJ/kg)	12.38	12.96
Corn gluten meal	5.37	5.80	Crude Protein	21.51	19.55
Soybean meal	33.99	28.33	Calcium	1.20	1.03
Soybean oil	3.30	5.19	Available Phosphate	0.46	0.42
Limestone	1.50	1.20	Lysine	1.32	1.19
CaHPO_4_	1.70	1.53	Methionine	0.54	0.51
NaCl	0.30	0.30	Methionine and Cysteine	0.93	0.88
DL-Methionine^A^	0.17	0.16	Zinc^E^ (mg/kg)	27.35	27.02
L-LYSiNE Monohydrochloride^B^	0.28	0.29			
Premix^C^	1.00	1.00			

A DL-Methionine, feed grade 99.0%.

B L-LYSiNE Monohydrochloride feed addition:content (C_6_H_14_N_2_O_2_.HCL, dry base meter ≥98.5%).

C The premix provided the following per kg of the diet:VA15200IU, VitD_3_ 4,400 IU, VE:32 IU VK_3_:3.2 mg, VB_1_4 mg, VB_2_12 mg, VB_6_4.8 mg, VB_12_0.032 mg, D-biotin 0.12 mg, Folic Acid 1.6 mg, nicotinamide 48 mg, D-pantothenic Acid 18 mg, Fe (Ferrous Sulfate) 100 mg, Cu (Copper Sulfate) 8 mg, Mn (Manganese Sulfate) 120 mg, I (Calcium Iodate) 0.7 mg, and Se (Sodium Selenite) 0.3 mg.

D Nutrient levels were calculated values.

E Values were determined by analysis, and each value was based on triplicate determinations.

### Production performance

On day 42, the body weight and feed consumption of the birds were recorded. Subsequently, the following parameters were calculated: average daily gain (ADG), average daily feed intake (ADFI), and the feed-to-gain (F: G) ratio. In addition, slaughter performance indicators and immune organ indices were systematically evaluated.

### Sample collection

On day 42 post-hatching, one broiler chicken, approximating the group mean body weight (±5%), was humanely euthanized via cervical dislocation from each replicate group. Immediate postmortem dissection was performed to collect tissue specimens within 3 min of euthanasia, in accordance with the AVMA Guidelines for the Euthanasia of Animals (2020 edition).

### Determination of trace element deposition and antioxidant capacity

The collected breast muscle, leg muscle, liver, tibia, and whole blood were stored at −20°C. Subsequently, the trace elements of Fe, Cu, Mn, and Zn in the tissues were determined using a flame atomic absorption spectrometer (Agilent Technology 200 Series AA, Malaysia) according to the NY/T3318-2018 standard recommended method. The liver and jejunum mucosal tissues were collected, frozen in liquid nitrogen, and stored at −80°C. Then, they were sent to Bio-company (Gansu Shuolian Biotechnology Co. Ltd., Gansu, China) for the determination of Cu/Zn-SOD activity, malondialdehyde (MDA) levels, and total antioxidant capacity (T-AOC) in the liver and jejunum using ELISA kits (Shanghai Enzyme Link Biotechnology Co., Ltd., Shanghai, China).

### Intestinal morphometry

After the broilers were slaughtered and dissected, tissue samples of approximately 1 ~ 2 cm were collected from the middle part of the duodenum, jejunum, and ileum. These samples were rinsed with normal saline and fixed in a paraformaldehyde solution. Then, these tissue blocks were sent to a biological company (Wuhan Seville Biotechnology Co., Ltd., Wuhan, China) to make a biopsy. Villus height (VH: from the tip of the villus to the villus–crypt junction) and crypt depth (CD: from the villus–crypt junction to the base of the crypt) were measured using a digital microscope (BA210 Digital, Motic China Group Co., Ltd., China). The ratio of villus height to crypt depth (VH/CD) was calculated at the same time.

### Relative mRNA expression levels of zinc-associated metal transporters in the jejunum

The mRNA expression levels of metal transporters—including Zn transporter 1 (ZnT-1), Zn transporter 5 (ZnT-5), metallothionein (MT), and divalent metal transporter 1 (DMT-1)—in the jejunal mucosa were determined using quantitative real-time PCR. Total RNA was extracted from the jejunal mucosa using TRIzol reagent (TransGen, China). The RNA was reverse transcribed into cDNA using the PrimeScript RT Reagent Kit (Takara, Japan) according to the manufacturer’s instructions. Real-time PCR was performed on a LightCycler 480 System (Roche, USA) using a SYBR Green Premix Pro Taq HS qPCR Kit (AG, China). The specific primers for the selected genes, listed in Table 2, were synthesized by Biotech (Suzhou Jin Wei Zhi Biological Technology Co., Ltd., China), with β-actin used as the internal reference. Differential gene expression was calculated using the 2^-△△Ct^ method.

**Table 2:** Primer sequences for real-time PCR amplification.

gene	Gene Bank ID	Forward primer (5′-3′)	Reverse primer (5′-3′)	Length (bps)
*ZnT-1*	AJ619980.1	TGCGAGTGCCTTCTTCCT	AAGGAGCTGTCAGGTCTGTAAT	131
*ZnT-5*	XM-424760.1	ATGCTGTTGTGGGATGTA	TTGTCTTGGCTGGTCCTC	159
*MT*	NM-205275.1	AAGGGCTGTGTCTGCAAGGA	CTTCATCGGTATGGAAGGTACAAA	163
*MT3*	NM-001097538.1	GACTGCCCTTGTGCCACC	TGCAGACGCAGCCCTGT	142
*DMT1*	NM-001128102.1	CGCACAATATGTACCTGCACTC	GCACGACTCGGCAAAGAAG	107
*MTF1*	NM-001031495.1	CCTGGTTCAACTCCTATGC	TCAAACGGCTTCTCCTTA	278

### Cecum microorganisms

Sequencing of the 16S rDNA was performed by GENE DENOVO (Guangdong, China). Illumina Novaseq 6,000 sequencing was used to characterize microbial diversity and community composition. Cecal microbial DNA was extracted using the HiPure Stool DNA Kits (Magen, Guangzhou, China) according to the manufacturer’s protocols. The 16S rDNA target region of the ribosomal RNA gene was amplified using PCR and primers located on both sides of the V3–V4 hypervariable region of the bacterial 16S rRNA gene. Related PCR reagents were obtained from TOYOBO (Japan). Amplicons were extracted from 2% agarose gels and purified using the AxyPrep DNA Gel Extraction Kit (Axygen Biosciences, Union City, CA, U.S.) according to the manufacturer’s instructions. The purified amplicons were then quantified using the ABI StepOnePlus Real-Time PCR System (Life Technologies, Foster City, USA).

Raw tags were filtered to obtain clean and high-quality tags, chimera sequences were removed, and sequences with 97% similarity were clustered into operational taxonomic units (OTUs) using QIIME2. Alpha diversity indices (Chao1, Shannon, and Simpson) and beta diversity of the cecal microbiota were calculated with QIIME2 and displayed with R software. Beta diversity was determined using the Bray–Curtis index and visualized through principal coordinates analysis (PCoA) and non-metric multidimensional scaling (NMDS) plots.

### Statistical analysis

All data were analyzed using SPSS 26.0 (IBM SPSS Software, Armonk, NY, USA), and statistical analysis was performed using one-way ANOVA with Duncan’s new multiple range test. The data were presented as the mean ± SD, and a *p*-value of < 0.05 was considered statistically significant. Among them, beta diversity analysis was performed using the Kruskal–Wallis H test and the analysis of similarities (ANOSIM) test.

## Results

### Production performance

The effects of Zn-MMT on growth performance, carcass parameters, and immune organ indices are shown in Table 3. Compared to the CK group, adding Zn-MMT to the diets did not significantly affect ADG, ADFI, thymus index, bursa index, and the percentages of slaughter, half-eviscerated, eviscerated, abdominal fat, pectoral muscle, leg, or leg muscle rate (*p* > 0.05). Compared to the CK group, Zn-MMT significantly reduced the F: G ratio and spleen index (*p* < 0.05).

**Table 3:** The effect of Zn-MMT on the production performance of the broilers.

Items		CK	Zn-MMT	ZnSO_4_	*p*-value
Growth performance	Average daily weight gain (ADG, g/d)	51.20 ± 1.99	50.77 ± 0.86	50.80 ± 3.68	0.948
	Average daily feed intake (ADFI, g/d)	88.63 ± 7.10	82.60 ± 2.65	87.35 ± 5.26	0.153
	Feed intake and weight gain ratio (F:G, g/g)	1.73 ± 0.04^a^	1.64 ± 0.04^b^	1.72 ± 0.03^a^	0.019
Carcass parameters	Slaughter percentage (%)	94.63 ± 1.24	93.46 ± 0.71	93.97 ± 0.70	0.121
	Half-eviscerated percentage (%)	86.19 ± 2.00	85.44 ± 0.61	85.35 ± 1.45	0.568
	Eviscerated percentage (%)	75.71 ± 5.21	73.83 ± 4.34	75.19 ± 1.45	0.708
	Abdominal fat percentage (%)	1.28 ± 0.22	1.16 ± 0.28	1.19 ± 0.34	0.758
	Pectoral muscle percentage (%)	33.54 ± 2.62^ab^	34.96 ± 1.92^a^	31.24 ± 1.29^b^	0.019
	Leg percentage (%)	31.99 ± 2.33	31.43 ± 2.91	30.17 ± 1.04	0.282
	Leg muscle percentage (%)	25.00 ± 2.19	25.10 ± 1.89	23.88 ± 1.27	0.458
Immune organ index	Thymus index (g/kg)	4.42 ± 0.72	4.63 ± 0.39	3.68 ± 0.48	0.080
	Bursa index (g/kg)	1.43 ± 0.36	1.52 ± 0.19	1.48 ± 0.48	0.935
	Spleen index (g/kg)	0.93 ± 0.11^a^	0.72 ± 0.87^b^	0.80 ± 0.11^ab^	0.035

Different letters in the same row mean significant differences between the treatments (*p* < 0.05), and the same letter or no letter in the same row means no significant differences between the treatments (*p* ≥ 0.05). The same applies below.

### Tissue trace element deposition

The effect of Zn-MMT on the deposition of the trace elements in the tissues is summarized in Table 4.

**Table 4:** The effects of Zn-MMT on trace element deposition in the tissues (mg/kg).

Items		CK	Zn-MMT	ZnSO_4_	*p*-value
Fe					
Day 21	Breast muscle	10.64 ± 1.39	8.19 ± 2.36	11.33 ± 4.42	0.197
	Leg muscle	14.55 ± 2.99	11.92 ± 2.68	15.17 ± 5.20	0.318
	Liver	142.99 ± 32.05	188.11 ± 62.72	152.21 ± 29.81	0.208
	Tibia	94.91 ± 61.10	108.70 ± 61.58	52.76 ± 24.08	0.186
	Pancreas	74.63 ± 25.14	66.11 ± 13.56	85.81 ± 46.71	0.569
	Whole blood	178.17 ± 58.72	157.04 ± 29.56	149.63 ± 19.29	0.451
Day 42	Breast muscle	2.66 ± 0.56^b^	9.69 ± 2.64^a^	3.66 ± 3.34^b^	<0.001
	Leg muscle	9.48 ± 5.15	7.85 ± 5.98	3.01 ± 0.34	0.067
	Liver	189.48 ± 11.92	168.07 ± 32.02	210.08 ± 42.21	0.100
	Tibia	49.64 ± 27.72	28.64 ± 12.85	29.33 ± 8.78	0.113
	Pancreas	64.85 ± 28.53	46.74 ± 11.53	48.48 ± 18.37	0.277
	Whole blood	173.01 ± 31.22	176.97 ± 40.52	187.50 ± 30.60	0.757
Cu					
Day 21	Breast muscle	0.96 ± 0.22	0.95 ± 0.17	1.13 ± 0.64	0.669
	Leg muscle	2.68 ± 1.30	1.38 ± 0.50	1.58 ± 0.69	0.050
	Liver	5.86 ± 0.93	5.37 ± 0.56	5.10 ± 0.81	0.266
	Tibia	8.53 ± 2.27	8.16 ± 2.05	6.77 ± 1.37	0.281
	Pancreas	8.53 ± 4.06	8.63 ± 2.97	7.34 ± 6.14	0.864
	Whole blood	0.64 ± 0.16	0.76 ± 0.27	0.73 ± 0.16	0.584
Day 42	Breast muscle	2.65 ± 1.04	3.00 ± 1.10	1.96 ± 1.16	0.283
	Leg muscle	3.50 ± 1.72	3.02 ± 0.92	3.43 ± 0.52	0.743
	Liver	2.36 ± 1.08^a^	2.89 ± 1.13^a^	1.43 ± 0.41^b^	0.046
	Tibia	5.20 ± 1.06	4.40 ± 0.80	4.45 ± 0.51	0.199
	Pancreas	3.58 ± 0.63	2.97 ± 0.62	3.06 ± 0.54	0.190
	Whole blood	0.89 ± 0.47	0.53 ± 0.18	0.74 ± 0.16	0.140
Mn					
Day 21	Breast muscle	1.06 ± 0.42	0.97 ± 0.42	1.03 ± 0.26	0.922
	Leg muscle	0.52 ± 0.25	0.61 ± 0.15	0.80 ± 0.37	0.228
	Liver	2.37 ± 0.53	2.24 ± 0.40	1.97 ± 0.31	0.266
	Tibia	6.95 ± 1.58	6.71 ± 1.34	5.84 ± 0.50	0.285
	Pancreas	4.50 ± 1.04	5.40 ± 0.53	4.03 ± 1.73	0.171
	Whole blood	0.50 ± 0.08	0.39 ± 0.07	0.39 ± 0.10	0.082
Day 42	Breast muscle	1.76 ± 1.50	2.07 ± 1.12	1.20 ± 0.59	0.421
	Leg muscle	2.15 ± 0.51	1.59 ± 0.50	1.87 ± 1.10	0.461
	Liver	4.82 ± 0.65^a^	5.39 ± 0.29^a^	4.05 ± 0.95^b^	0.014
	Tibia	5.33 ± 1.06	4.82 ± 1.74	4.64 ± 0.94	0.639
	Pancreas	4.02 ± 1.44	4.97 ± 3.04	4.05 ± 0.86	0.657
	Whole blood	0.36 ± 0.14	0.26 ± 0.11	0.23 ± 0.04	0.091
Zn					
Day 21	Breast muscle	8.57 ± 2.20	7.50 ± 0.26	6.62 ± 1.52	0.127
	Leg muscle	15.22 ± 4.77	15.92 ± 3.75	13.84 ± 3.39	0.668
	Liver	50.20 ± 13.23	50.09 ± 25.72	52.72 ± 17.25	0.966
	Tibia	138.84 ± 20.11^a^	157.24 ± 13.75^a^	124.2 ± 10.75^b^	0.007
	Pancreas	52.34 ± 25.18^b^	113.33 ± 16.67^a^	46.93 ± 18.57^b^	<0.001
	Whole blood	4.87 ± 0.88	4.13 ± 0.96	3.46 ± 1.16	0.084
Day 42	Breast muscle	9.98 ± 1.40	8.95 ± 1.10	8.47 ± 0.61	0.081
	Leg muscle	11.42 ± 1.91	11.98 ± 6.49	17.58 ± 4.41	0.069
	Liver	10.67 ± 1.90^b^	55.1 ± 19.04^a^	9.69 ± 2.27^b^	<0.001
	Tibia	54.88 ± 9.04^b^	78.6 ± 8.09^a^	75.91 ± 12.93^a^	0.002
	Pancreas	38.85 ± 15.46^b^	103.75 ± 17.96^a^	56.33 ± 10.79^b^	<0.001
	Whole blood	4.55 ± 1.51^b^	6.72 ± 2.22^a^	7.94 ± 0.88^a^	0.009

In terms of the Fe content, throughout the entire experimental period, except for the Fe content in the breast muscle at 42 days of age, the addition of Zn had no significant (*p* > 0.05) impact on the iron content in the pectoral muscle, leg muscle, liver, tibia, pancreas, and whole blood. Compared to the CK and ZnSO_4_ groups, adding Zn-MMT to the diets significantly increased (*p* < 0.05) the Fe content in the breast muscle at 42 days of age, and there was no significant difference between the CK group and the ZnSO_4_ group (*p* > 0.05). In terms of the content of Cu and Mn, similar trends were observed. Compared to the CK group, the addition of Zn had no effect (*p* > 0.05) on the content of Cu and Mn in all tissues and blood, except in the liver at 42 days of age. Compared to the CK group, ZnSO_4_ significantly decreased (*p* < 0.05) the levels of Cu and Mn in the liver at 42 days of age. As for the content of Zn, at 21 days of age, compared to the CK and ZnSO_4_ groups, the addition of Zn-MMT to the diet significantly increased (*p* < 0.05) the pancreatic Zn content. However, dietary ZnSO_4_ supplementation decreased (*p* < 0 0.05) the tibia Zn content compared to the CK and Zn-MMT groups. At 42 days of age, Zn-MMT supplementation significantly (*p* < 0.05) affected the pancreatic and liver Zn levels, compared to the CK group, and Zn-MMT notably increased (*p* < 0.05) the pancreatic Zn content. Compared to the CK group, Zn supplementation significantly increased (*p* < 0.05) the Zn content in the tibia and whole blood.

### Antioxidant capacity

The effects of Zn-MMT on antioxidant capacity are summarized in Table 5. Compared to the CK group, adding Zn-MMT and ZnSO_4_ to the diets significantly increased (*p* < 0.05) T-AOC and Cu/Zn-SOD in the liver and jejunum. In contrast, Zn could significantly reduce the MDA content in the liver and jejunum (*p* < 0.05). Simultaneously, ZnSO_4_ was more effective than Zn-MMT in reducing the MDA content in the liver and jejunal mucosa (*p* < 0.05).

**Table 5:** The effects of Zn-MMT on the antioxidant capacity of the liver and jejunal mucosa in the broilers.

Items		CK	Zn-MMT	ZnSO_4_	*p*-value
Day 21					
Liver	MDA (nmol/ml)	3.29 ± 0.14^a^	3.02 ± 0.10^b^	2.12 ± 0.11^c^	<0.001
	T-AOC (U/ml)	4.83 ± 0.20^b^	6.19 ± 0.20^a^	6.39 ± 0.32^a^	<0.001
	Cu Zn-SOD (U/ml)	123.34 ± 3.94^b^	148.95 ± 3.61^a^	152.69 ± 5.84^a^	<0.001
Jejunum mucosa	MDA (nmol/ml)	4.60 ± 0.20^a^	4.22 ± 0.14^b^	2.93 ± 0.15^c^	<0.001
	T-AOC (U/ml)	4.42 ± 0.19^b^	5.69 ± 0.19^a^	5.89 ± 0.30^a^	<0.001
	Cu Zn-SOD (U/ml)	127.01 ± 4.06^b^	153.38 ± 3.72^a^	157.23 ± 6.02^a^	<0.001
Day 42					
Liver	MDA (nmol/ml)	3.57 ± 0.14^a^	3.30 ± 0.09^b^	2.40 ± 0.11^c^	<0.001
	T-AOC (U/ml)	5.68 ± 0.24^b^	7.30 ± 0.23^a^	7.54 ± 0.38^a^	<0.001
	Cu Zn-SOD (U/ml)	114.07 ± 3.65^b^	137.76 ± 3.34^a^	141.21 ± 5.40^a^	<0.001
Jejunum mucosa	MDA (nmol/ml)	5.26 ± 0.21^a^	4.85 ± 0.14^b^	3.49 ± 0.16^c^	<0.001
	T-AOC (U/ml)	4.15 ± 0.18^b^	5.37 ± 0.18^a^	5.55 ± 0.29^a^	<0.001
	Cu Zn-SOD (U/ml)	99.02 ± 3.18^b^	119.68 ± 2.91^a^	122.69 ± 4.71^a^	<0.001

### Intestinal morphology

The effects of Zn-MMT on intestinal morphology are summarized in Table 6. Compared to the CK group, adding Zn-MMT to the diets significantly (*p* < 0.05) increased VH and the VH/CD ratio in the duodenum and jejunum, as well as increased CD in the jejunum. Simultaneously, compared to the CK group, Zn-MMT decreased CD in the duodenum. Compared to the CK group, dietary supplementation with Zn-MMT or ZnSO_4_ significantly (*p* < 0.05) increased VH and the VH/CD ratio in the ileum. Ileum CD was not significantly (*p* > 0.05) affected by the addition of Zn from different sources.

**Table 6:** The effect of Zn-MMT on the intestinal morphology of the broilers.

Items		CK	Zn-MMT	ZnSO_4_	*p*-value
Duodenum	VH/μm	909.90 ± 99.13^b^	1125.20 ± 40.02^a^	868.25 ± 50.21^b^	<0.001
	CD/μm	159.15 ± 12.43^a^	140.22 ± 3.61^b^	160.40 ± 8.00^a^	0.003
	VH/CD	5.41 ± 0.55^b^	7.93 ± 0.74^a^	5.90 ± 0.59^b^	<0.001
Jejunum	VH/μm	886.02 ± 162.55^b^	1121.28 ± 55.77^a^	869.01 ± 56.53^b^	<0.001
	CD/μm	116.74 ± 9.43^b^	134.57 ± 9.03^a^	119.13 ± 16.90^b^	0.028
	VH/CD	7.82 ± 0.42^b^	8.68 ± 0.43^a^	7.61 ± 0.95^b^	0.034
Ileum	VH/μm	655.87 ± 84.50^b^	787.49 ± 39.23^a^	714.71 ± 49.33^ab^	<0.001
	CD/μm	136.51 ± 20.60	130.48 ± 14.97	122.65 ± 15.91	0.352
	VH/CD	4.92 ± 0.67^b^	6.38 ± 0.59^a^	5.93 ± 0.66^a^	0.002

### Relative mRNA expression of metal transporters in the jejunum

**Figure 1:**
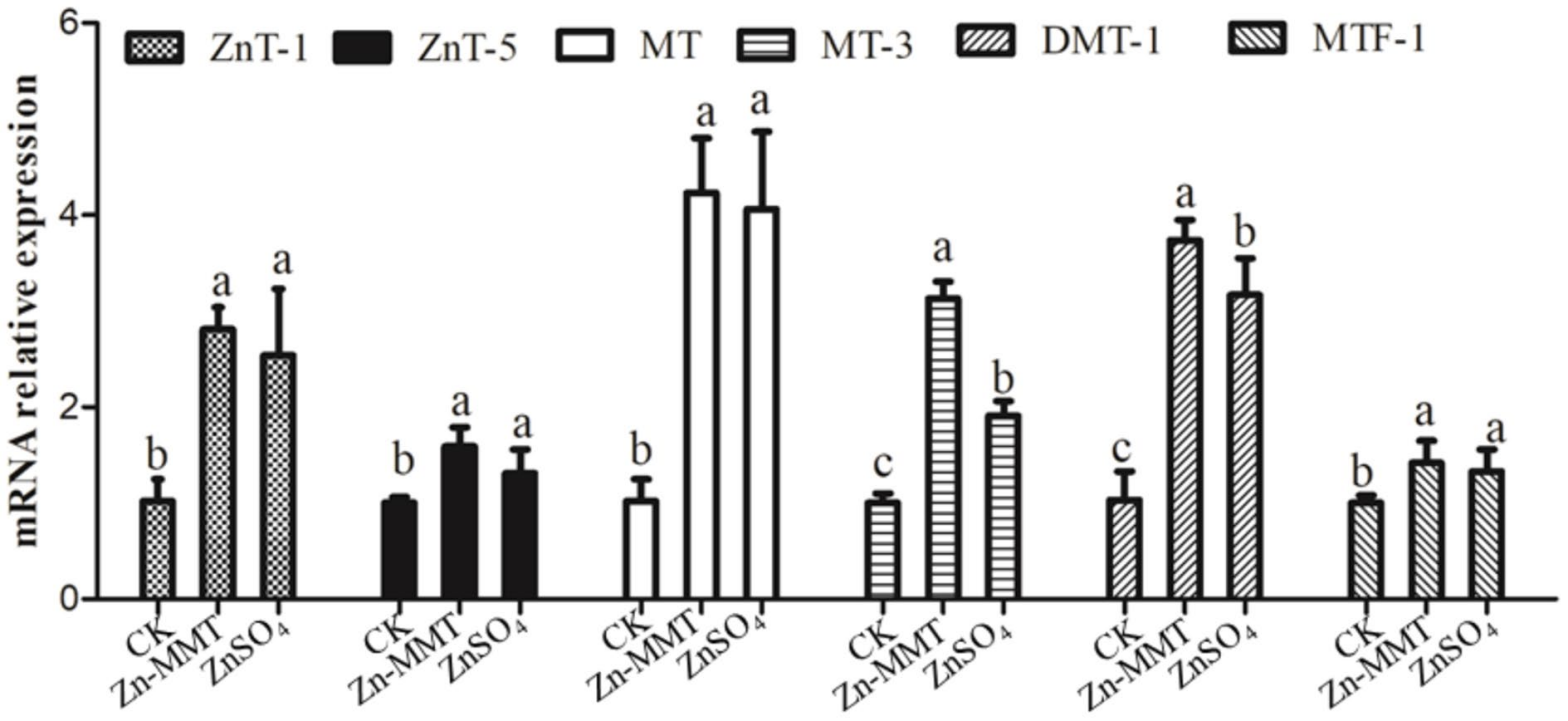
Effects of Zn-MMT on metal transport carriers in the jejunum of the broilers. The data shown represent the mean ± SD of at least three independent experiments, and different lowercase letters indicate significant differences (*p* < 0.05).

The effects of Zn-MMT on jejunum metal transporter relative mRNA expression are summarized in Figure 1. Compared to the CK group, adding Zn-MMT and ZnSO_4_ to the diets significantly (*p* < 0.05) increased the mRNA relative expression of *ZnT-1, ZnT-5*, *MT*, and *MTF-1* in the jejunum. Compared to the CK group, adding Zn-MMT and ZnSO_4_ to the diets significantly increased the mRNA relative expression of *MT-3* and *DMT-1*. As for the mRNA expression of *MT-3* and *DMT-1* in the jejunum, the addition of Zn-MMT was significantly greater than that of ZnSO_4_ (*p* < 0.05).

### Cecum microbiome

**Figure 2:**
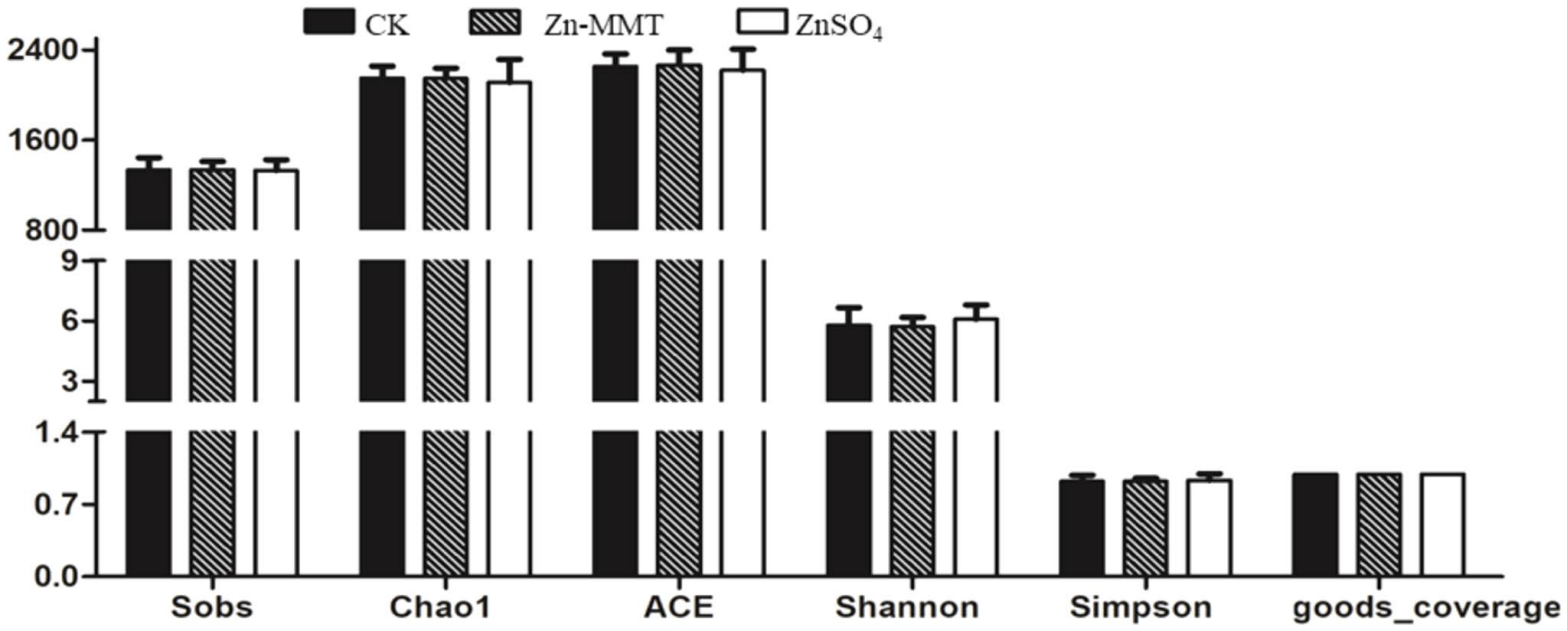
Determination of bacterial community α-diversity in the cecum of the broilers. The figure includes the Observed species, Chao1 index, ACE index, Shannon index, Simpson index, and Goods_coverage, with the CK, Zn-MMT, and ZnSO₄ groups representing the control, Zn-MMT, and ZnSO₄ treatments, respectively. Unmarked letters on the bar chart indicate no significant difference between the groups.

**Figure 3:**
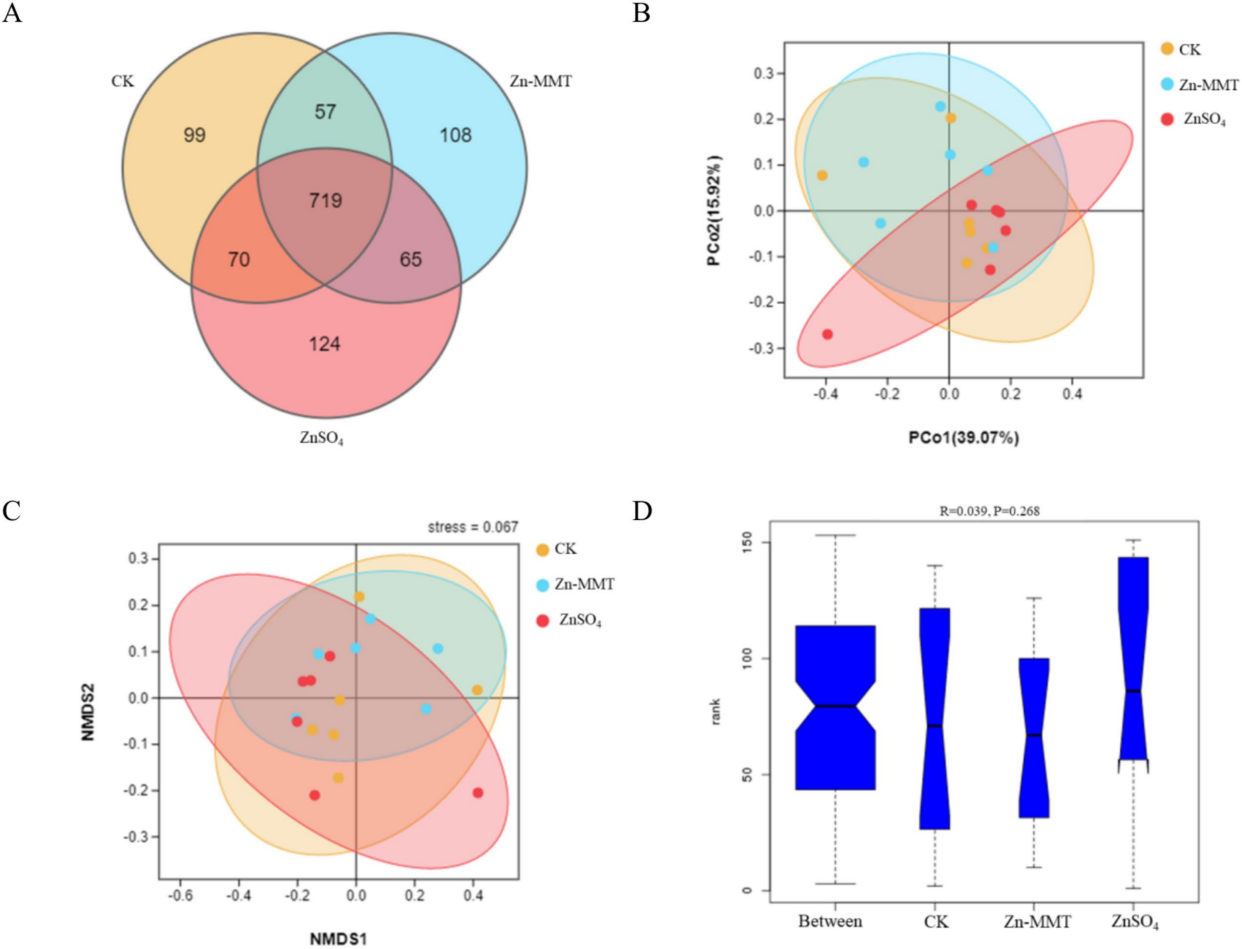
Comparison of gut bacterial communities among the CK, MMT, and Zn groups. The Venn diagram shows the shared operational taxonomic units (OTUs) in each group. The overlapping part refers to the amount of OTUs shared between the groups, while the non-overlapping part refers to the only amount of OTUs in the group **(A)**. Principal coordinates analysis (PCoA) **(B)**, Non-metric multidimensional scaling (NMDS) analysis **(C)**, and analysis of similarities (ANOSIM) **(D)**. CK = control group; MMT = Zn-loaded MMT group; Zn = ZnSO_4_ group.

**Figure 4:**
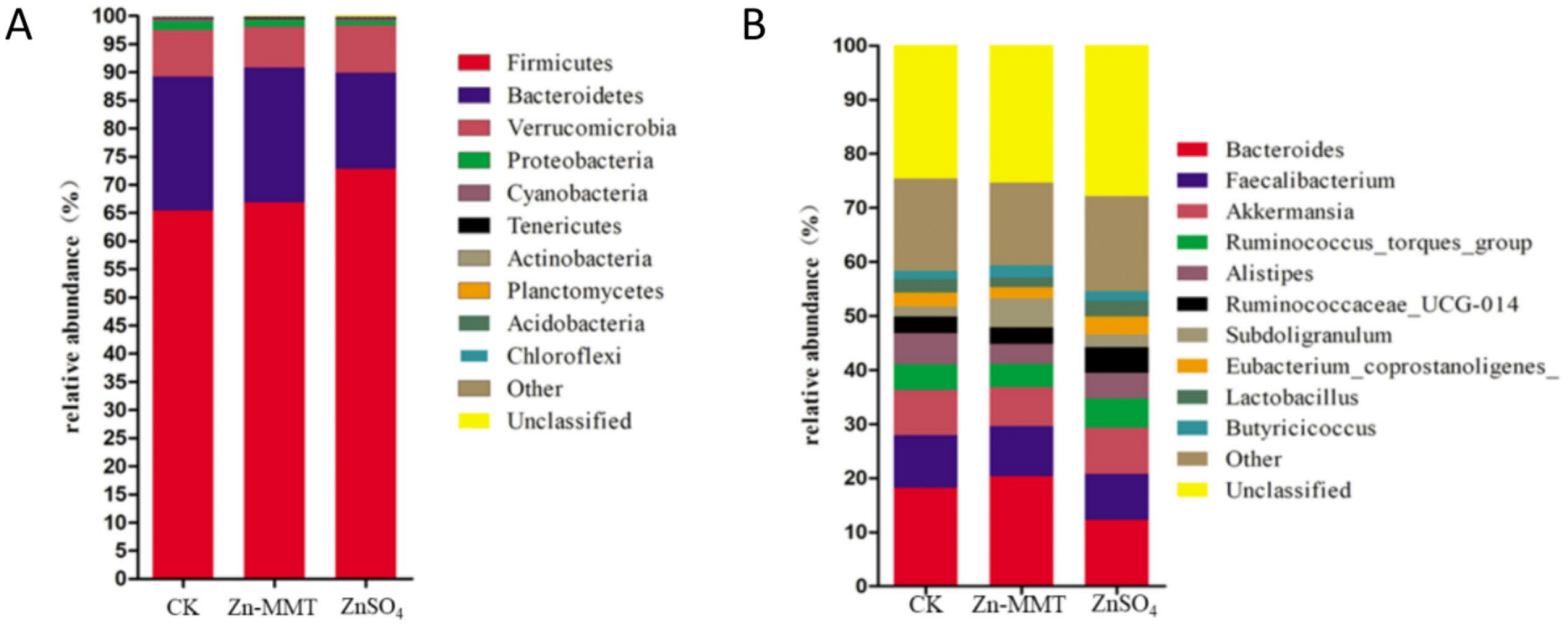
The relative abundance of the top 10 phyla **(A)** and the top 10 genera **(B)** of the gut bacteria.

The effects of Zn-MMT on the alpha and beta diversity of the cecum microbiome in the broilers are shown in Figures 2, 3, respectively. As shown in Figure 2, Zn-MMT had no significant effect on the observed species, Shannon index, Simpson index, ACE index, or Chao1 index (*p* > 0.05). The results of PCOA, NMDS, and ANOSIM were consistent, and there was no independent distribution among the treatment groups, indicating that there was no significant difference in the structure and diversity of the cecal bacteria in the broilers. A total of 17 phyla and 214 genera were identified in the cecum microorganisms. The 10 most abundant phyla and genera are presented in Figure 4. At the phylum level (Figure 4A) and at the genus level (Figure 4B), the top 10 bacterial genera in the CK, Zn-MMT, and ZnSO_4_ groups accounted for more than 75.58% of the annotated genera. However, at the phylum and genus levels, the abundance of the top 10 bacterial genera among the treatment groups was not significantly different.

## Discussion

### Effects of Zn-MMT on growth performance

The chemical form of Zn plays a critical role in determining its bioavailability and biological functions in broilers, thereby significantly influencing growth performance (18–20). Among various mineral delivery systems, MMT-intercalated trace elements have emerged as a promising strategy. As an efficient nutrient carrier, MMT offers unique advantages in sustained nutrient release. Hu et al. (16) reported that ZnO-MMT significantly enhanced broiler growth compared to conventional ZnO supplementation. Similarly, Jiao et al. (21) demonstrated that dietary supplementation with 150 mg/kg Zn in the form of Zn-MMT improved production performance in weaned pigs. Building on this approach, Jiao et al. (22) further revealed that Cu/Zn-MMT supplementation in weaned piglet diets resulted in superior growth performance compared to inorganic Cu and Zn sources. Notably, Eckhardt et al. (23) documented that the administration of calcium-modified MMT (Ca-MMT) not only increased body weight but also enhanced daily feed intake and feed conversion efficiency in poultry. In the present study, neither the ADG nor ADFI of the broilers showed significant differences among the experimental groups supplemented with Zn-MMT or ZnSO₄. The NRC set the Zn requirement at a level of 40 mg/kg for broilers. Corn–soybean meal-based diets typically contain Zn concentrations exceeding 40 mg/kg (20), which may adequately satisfy the growth demands of chicks and thereby explain the absence of significant effects on weight gain.

The feed conversion ratio (F: G) was significantly influenced by Zn-MMT supplementation. The superior performance associated with Zn-MMT could be attributed to synergistic interactions between the montmorillonite matrix and Zn ions, which may enhance nutrient utilization efficiency through improved mineral bioavailability and intestinal absorption capacity (16, 18). The intestinal mucosal protective mechanisms of montmorillonite (MMT) operate through two primary pathways: (1) MMT exhibits selective adsorption capacity through its layered nanostructure and cation exchange properties (24), and (2) MMT interacts with mucin glycoproteins to enhance the physicochemical properties of the mucus layer (25). As an essential cofactor for over 300 metalloenzymes, including alkaline phosphatase, carbonic anhydrase, and matrix metalloproteinases, Zn orchestrates critical biological processes, ranging from nucleic acid metabolism to antioxidant defense systems (26). Its bacteriostatic activity arises from the competitive inhibition of microbial magnesium uptake and the disruption of bacterial membrane potential, particularly against enteropathogenic *Escherichia coli* and *Salmonella* spp. (1). The interlayer spacing and high specific surface area render MMT an exceptional controlled-release carrier (13, 15, 16). Zn ions can be intercalated into MMT’s octahedral sheets through ion-exchange reactions, achieving sustained release kinetics that correlate with gastrointestinal pH variations. This pH-responsive release profile optimizes Zn bioavailability while minimizing ionic oversaturation toxicity. It has been hypothesized that the clinoptilolite component in Zn-MMT functions as a sustained-release carrier, potentially modulating the temporal and spatial release kinetics of Zn within the gastrointestinal tract. This controlled-release mechanism may enhance Zn bioavailability by prolonging its intestinal retention time and facilitating targeted delivery to the hindgut region, thereby amplifying its biological efficacy and antimicrobial activity (16, 27). Subsequent studies have further demonstrated that the MMT matrix in Zn-MMT creates ion-exchange channels that regulate Zn dissolution rates while protecting against premature degradation in the proximal gut (28, 29). Such optimized Zn delivery characteristics not only improve intestinal zinc absorption but also potentiate its bacteriostatic effects in the colonic environment. Collectively, these mechanisms contribute to enhanced gastrointestinal homeostasis and improved growth performance in broilers. Notably, the supplementation of Zn-MMT reduced the spleen index in the present trial, which contrasts with previous findings (30). As the largest immune organ, the spleen serves as a central hub for both cellular and humoral immunity, playing a crucial role in immune regulation. The precise mechanisms underlying this phenomenon warrant further investigation.

### Effects of Zn-MMT on mineral retention in the tissues

In the study, the supplementation of Zn-MMT increased the Zn concentration in the liver, tibia, pancreas, and whole blood. It is well established that the deposition of trace minerals in various tissues and organs is an important parameter for the normal growth and development of broilers. The above results are consistent with the observed growth performance of the broilers in this experiment, suggesting that Zn-MMT can be used as a Zn supplement in broiler feed and that it may be better than ZnSO_4_. Research has reported that Zn in Zn-MMT has superior bioavailability, which may be related to the controlled-release properties of MMT. MMT can adsorb nutrients and gradually control their release, thereby increasing their duration of action or biological effects. These results indicate that Zn-MMT may alter the rate, timing, or location of Zn release, thereby improving the biological effects of Zn. This modulation is beneficial for Zn accumulation, tissue or organ development, and overall growth performance in broilers. The dietary inclusion of Zn-MMT significantly increased Zn concentrations in key metabolic tissues, including the liver, tibia, pancreas, and whole blood, compared to the control group. Tissue-specific Zn deposition serves as a critical biomarker for evaluating trace mineral bioavailability and metabolic homeostasis in broilers (31). These findings align with the observed improvements in growth performance parameters, suggesting that Zn-MMT functions as an effective zinc supplement in broiler nutrition. Notably, the enhanced bioavailability of Zn-MMT compared to conventional ZnSO_4_ may be attributed to montmorillonite’s unique cation-exchange capacity and layered structure (16). As demonstrated by Tang et al. (32), the interlamellar spaces of MMT enable the controlled release of adsorbed Zn ions through a pH-dependent mechanism, prolonging nutrient retention time in the gastrointestinal tract. This sustained-release property reduces ionic competition with dietary antagonists, thereby improving Zn absorption efficiency compared to ZnSO_4_ (33, 34). Similar findings were reported by Huang et al. (20), who demonstrated that dietary Zn-MMT supplementation elevated Zn concentrations in biological specimens. This observation was corroborated by Tang et al. (18), whose investigation revealed that Zn-loaded zeolite supplementation significantly enhanced Zn deposition in the pancreas, liver, and tibia of broilers. Jiao et al. (22) reported that the inclusion of Cu/Zn-MMT increased Zn concentrations in the jejunal and ileal mucosa compared to controls, suggesting that the Zn release *in vivo* was prolonged. Current research on the impact of Zn-MMT on Fe, Mn, and Cu homeostasis remains limited. In laying hens, Li et al. (34) observed that Zn-bearing zeolite supplementation increased serum Fe levels. Furthermore, Yang et al. (33) identified a linear increase in the Fe content with dietary supplementation of Zn-palygorskite. In the current study, dietary Zn-MMT supplementation significantly influenced the Fe content in the breast muscle of the broilers, which aligns with previous observations in avian species. However, this finding contrasts with data from Jiao et al. (22), who reported no alterations in the Fe concentration across tissues and feces when supplementing Cu/Zn-MMT in weaned piglets. In this study, we observed that dietary ZnSO_4_ supplementation significantly reduced Mn and Cu concentrations in the liver compared to the CK and Zn-MMT groups. Notably, synergistic and antagonistic interactions between trace elements are reported to directly influence the absorption and metabolism of essential minerals in animals (35). Given the conflicting reports regarding the effects of Zn supplementation on trace element concentrations in biological matrices, further investigation is required to elucidate how different dietary Zn sources affect trace element absorption, metabolism, and accumulation in animals.

### Effects of Zn-MMT on antioxidant capacity and intestinal morphology

Certain trace elements (Cu, Zn, and Mn) serve as structural components of metalloenzymes that scavenge reactive oxygen species and mitigate oxidative damage. Zn is constitutively required for maintaining the structural integrity and catalytic activity of Cu/Zn superoxide dismutase (Cu/Zn SOD). As the predominant SOD isoform, accounting for 90% of total SOD activity, Cu/Zn SOD provides essential protection against oxidative injury in vulnerable tissues such as the nervous and pulmonary systems (36). This biochemical dependency suggests that modulation of tissue Zn concentrations may directly influence antioxidant capacity. The experimental results demonstrated that dietary Zn-MMT supplementation significantly enhanced antioxidant capacity in the broilers compared to conventional ZnSO₄ supplementation. Notably, the broilers receiving Zn-MMT showed substantial improvements in antioxidant parameters: total antioxidant capacity (T-AOC) increased by 28.5% in the liver and 29.4% in the jejunal mucosal tissue, while Cu/Zn-superoxide dismutase (Cu/Zn-SOD) activity exhibited significant elevation. These observations align with previous reports by Yang et al. (33) and Xie et al. (37) on Zn’s antioxidant properties. This effect was further corroborated by Dukare et al. (38), who documented increased serum SOD levels following 80 mg/kg Zn supplementation. Interestingly, we observed that dietary supplementation with Zn-MMT and ZnSO₄ significantly reduced the MDA content in the liver and jejunum, with the ZnSO₄ group showing significantly lower levels than the Zn-MMT group. This phenomenon may be attributed to the aldehyde and carbonyl groups in MDA forming stable complexes with Zn^2+^ ions from ZnSO₄, thereby reducing MDA accumulation. The distinct efficacy of ZnSO₄ compared to Zn-MMT likely stems from differences in zinc bioavailability or chemical interactions. ZnSO₄ dissociates more readily *in vivo*, releasing free Zn^2+^ for chelation with MDA, whereas Zn-MMT may exhibit slower ion release kinetics, partially limiting its capacity to neutralize MDA. These findings indicate that dietary Zn supplementation enhances the antioxidant capacity of broilers, with Zn-MMT demonstrating superior efficacy in activating antioxidant enzyme systems compared to ZnSO_4_ and thereby alleviating oxidative stress-induced cellular damage.

Oxidative stress has been extensively documented to exert significant interactions with intestinal morphology. As a critical organ system, the intestinal tract plays an essential role in nutrient digestion, absorption, and immune regulation. VH, CD, and their ratio (VH/CD) serve as key morphological indicators for assessing intestinal integrity and nutrient assimilation capacity. Zn, an essential trace mineral, has been shown to modulate intestinal morphology, improve nutrient absorption efficiency, and enhance growth performance through multiple physiological mechanisms. Previous studies have demonstrated that dietary Zn supplementation enhances VH and the VH/CD ratio in the small intestine of broilers, although the efficacy varies significantly depending on the Zn source (1, 5). Our study demonstrated that dietary supplementation with Zn-MMT significantly increased VH and the VH/CD ratio in the intestinal mucosa by an average of 23.4 and 25.8%, respectively. These findings align with the observations reported by Tang et al. (31) in chicks and Jiao et al. (21) in weaned piglets. Zn-MMT demonstrated superior efficacy compared to ZnSO_4_ in improving VH and the VH/CD ratio while also significantly reducing crypt depth. Emerging evidence indicates that Zn plays a pivotal role in intestinal epithelial repair and the maintenance of mucosal integrity through three primary mechanisms (1): stabilization of tight junction proteins (2); modulation of inflammatory cytokine expression (3); enhancement of antioxidant enzyme activity. The potential mechanism may involve Zn’s regulatory effects on DNA and protein synthesis, inhibition of cellular apoptosis, and modulation of cell proliferation (39). MMT interacts with gastrointestinal mucus proteins through selective binding, which enhances mucus secretion while improving its cohesive and elastic properties, ultimately promoting mucosal integrity protection and repair (40). This suggests that Zn-MMT’s protective effects on the intestinal barrier likely result from synergistic interactions between Zn and the components of MMT.

### Effects of Zn-MMT on relative mRNA expression of metal transporters in the jejunum

Zn homeostasis is predominantly regulated through the coordinated modulation of intestinal Zn absorption, transport, and excretion. Studies have demonstrated that Zn absorption in the animal intestine occurs via two distinct mechanisms: passive diffusion and saturable, carrier-mediated transport. The latter represents an energy-dependent process facilitated by specific Zn-binding transporter proteins. The carrier-mediated Zn absorption pathway in the intestinal epithelium can be categorized into three sequential steps: (1) Apical uptake: Zn ions traverse the apical membrane of enterocytes from the intestinal lumen into the cytoplasm, mediated by transporters such as Zrt-/Irt-like protein. (2) Intracellular trafficking: Zn undergoes cytosolic redistribution from the apical to the basolateral membrane, involving metallothionein buffering and vesicular transport mechanisms. (3) Basolateral efflux: Zn is extruded across the basolateral membrane into systemic circulation via *ZnT*, completing its transcellular translocation. Zn homeostasis in animals is regulated by multiple transport carriers, including *ZnT*, *DMT-1*, *MT*, and *MTF-1* (41). Current evidence demonstrates that *ZnT-1* and *ZnT-5* primarily mediate Zn ion absorption and transport in the duodenal and jejunal epithelia, facilitating the translocation of Zn^2+^ from the cytoplasm to peripheral circulation or intracellular organelles (42, 43). MT, a cysteine-rich, low-molecular-weight protein, exhibits a high binding affinity for both zinc and heavy metals (44). Although *DMT-1* participates in the transport of divalent cations, its Zn translocation capacity operates independently of other cation transport systems (45–47). In broilers, Cao et al. (48) demonstrated that Zn-enriched diets upregulated *MT* mRNA levels in both pancreatic and hepatic tissues. This finding supports earlier observations in rats, where *MT* expression in the liver increased in a dose-dependent manner in response to dietary Zn intake (44). Similar regulatory patterns have been documented in weaned piglets (49) and poultry (50), confirming the evolutionary conservation of Zn-mediated *MT* modulation. These coordinated responses in *MT* and *ZnT-1* expression highlight their synergistic roles in Zn absorption and detoxification, although the precise molecular mechanisms warrant further investigation. In this study, dietary supplementation with 40 mg/kg Zn, either as Zn-MMT or ZnSO_4_, in the corn–soybean meal diets elicited significant upregulation of jejunal Zn transport machinery. Both treatments increased the mRNA levels of Zn-specific transporters (ZnT-1: 2.8-fold, ZnT-5: 1.6-fold) and metallothionein isoforms (MT: 4.1-fold, MT-3: 3.1-fold) compared to the control group. Notably, Zn-MMT supplementation demonstrated 18% greater induction efficacy for DMT-1 expression and 63.4% higher MT-3 levels compared to the ZnSO_4_ treatment. The coordinated upregulation of MTF-1 (1.42-fold) suggests enhanced metal-responsive element binding activity under Zn-MMT exposure. The observed discrepancy may be attributed to differential absorption and transport regulation mechanisms between ZnSO_4_ and Zn-MMT in the intestine. The sustained-release properties of Zn-MMT enable gradual Zn liberation within the intestinal tract, effectively maintaining stable luminal Zn concentrations while enhancing the expression of Zn transport-related proteins. However, the specific underlying mechanisms warrant further systematic investigation to elucidate the precise regulatory pathways involved. These findings provide a mechanistic basis for the observed improvements in Zn accumulation in the tibia and whole blood of 42-day-old broilers. The enhanced mineral retention may be attributed to the unique properties of metal-modified clays, as prior studies have reported that palygorskite (51), clinoptilolite (52), Ca-MMT (53), and Cu-MMT (14) can optimize intestinal morphology, modulate metal transporter expression, and consequently improve nutrient absorption and transport efficiency.

### Effects of Zn-MMT on the cecum microbiome

Through comprehensive α- and β-diversity analyses, we observed that key microbial community parameters—including total species richness (α-diversity), between-sample diversity patterns (β-diversity), and species distribution evenness—remained comparable across all treatment groups. The Firmicutes, Bacteroidetes, and Verrucomicrobia phyla collectively accounted for over 97% of the cecal microbial community in the broilers. At the genus level, this phylum-level dominance was further reflected in the prevalence of three core microbial taxa: *Bacteroides* (Bacteroidetes phylum), *Faecalibacterium* (Firmicutes phylum), and *Akkermansia* (Verrucomicrobia phylum). Furthermore, no significant differences were observed in the relative abundance of the top 10 bacterial genera at both phylum and genus levels. A previous study by Hu et al. (16) demonstrated that ZnO-MMT significantly reduced *Clostridium* spp. populations in both the small intestine and the cecum of broilers. In contrast to these findings, our study revealed no significant differences in cecal microbial composition between the broilers who received Zn-MMT and those supplemented with ZnSO_4_. These findings align with the established patterns of dominant gastrointestinal microbiota composition in terrestrial vertebrates (54). Although MMT exhibits adsorption capacity toward certain intestinal bacteria, its interaction may not induce substantial alterations in gut microbiota composition at higher taxonomic levels (phylum and genus), which could be attributed to the resilient symbiotic equilibrium maintained between the host and gut microbiota (55).

## Conclusion

In conclusion, Zn-MMT supplementation showed multifaceted beneficial effects in the broilers. Specifically, it enhanced intestinal morphological development by improving the VH/CD ratio and epithelial integrity. Furthermore, Zn-MMT upregulated the expression of key metal transporters (ZnT-1 and ZnT-5) in duodenal enterocytes, thereby promoting systemic Zn deposition and utilization efficiency. Notably, the intervention group exhibited enhanced antioxidant defense mechanisms through increased SOD activity, along with reduced MDA levels in the intestinal tissues. These findings collectively suggest that Zn-MMT serves as an effective Zn supplement that synergistically improves intestinal health parameters, optimizes Zn metabolic pathways, and reinforces antioxidant capacity in commercial broiler production.

## Data Availability

Sequence data that support the findings of this study have been deposited in the NCBI Sequence Read Archive (SRA) under the accession number: PRJNA1267805, access link: https://www.ncbi.nlm.nih.gov/bioproject/PRJNA1267805.
